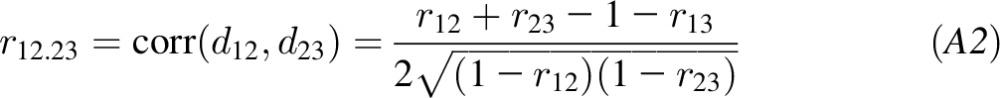# Weight centile crossing in infancy: correlations between successive months show evidence of growth feedback and an infant-child growth transition^1^

**DOI:** 10.3945/ajcn.116.139774

**Published:** 2016-09-07

**Authors:** Tim J Cole, Atul Singhal, Mary S Fewtrell, Jonathan CK Wells

**Affiliations:** Population, Policy, and Practice Program, University College London Great Ormond Street Institute of Child Health, London, United Kingdom

**Keywords:** growth, weight, centile crossing, infancy, feedback

## Abstract

**Background:** Early rapid weight gain is associated with later overweight, which implies that weight centile crossing tracks over time.

**Objective:** Centile crossing is defined in terms of the change or deviation in weight *z* score during 1 mo, and the correlations between successive deviations are explored at different ages.

**Design:** Two Cambridge (United Kingdom) growth cohorts were used: Widdowson (1094 infants born during 1959–1965) and the Cambridge Infant Growth Study (CIGS; 255 infants born during 1984–1987), each with weights measured monthly in the first year. Weights were converted to WHO age- and sex-adjusted *z* scores, deviations were calculated as the change in *z* score between adjacent measurement occasions, and the correlations between deviations were studied.

**Results:** In both cohorts, the correlations between successive monthly deviations were positive in the first 6 mo and highest at ages 3–4 mo (*r* = 0.3, *P* < 0.0001), whereas after 6 mo they were negative and were lowest at ages 10–11 mo (*r* = –0.3, *P* < 0.0001), with the correlation decreasing linearly with age between these extremes. Thus, during the first 6 mo of age, infants crossing centiles in 1 mo tended to continue crossing centiles in the same direction the following month, whereas after 6 mo they tended to cross back again. This represents positive and negative feedback, respectively. At age 12 mo, the correlation was close to zero, which suggests an infant-child transition in growth.

**Conclusions:** The results confirm that weight centile crossing tracks over time, with the correlations between successive periods that change with age suggesting a complex feedback mechanism underlying infant growth. This may throw light on the link between early rapid weight gain and later overweight. Clinically, the correlations indicate that when predicting future weight from current weight, recent centile crossing affects the prediction in an age-dependent manner.

## INTRODUCTION

In childhood, growth is well established to be canalized or “self-correcting” under the genetic influence of growth hormone (1). In infancy, in contrast, growth is under nutritional regulation and is more plastic (2, 3). Little is known about the tendency for growth to “track” during such early critical windows of plasticity.

As an example, the growth acceleration hypothesis (4) suggests that weight gain in early infancy correlates positively with lean mass and fat mass later in childhood (5–9). If defined as the change or deviation in *z* score over time, rapid early weight gain equates to a positive deviation or upward centile crossing on the weight chart. The risk of being relatively heavier later then depends on the subsequent pattern of centile crossing. Do infants who have crossed to a higher centile tend to stay there, or do they cross even higher, or do they instead tend to regress to a lower centile? What is the correlation between centile crossing (i.e., between deviations) in successive time intervals?

The concept of tracking shows itself as highly correlated serial measurements, usually after conversion to *z* scores based on a suitable growth reference (10). Calculating the weight *z* score correlation structure in terms of pairs of measurement ages allows weight gain over time to be expressed as a velocity *z* score, which is either unconditional or conditional on the first weight (11). The correlation between successive monthly weight *z* scores increases with age, from ∼0.8 at 0–1 mo to >0.97 at 11–12 mo (10–12).

However, the interest here is in acceleration rather than velocity (i.e., the change in centile crossing or deviation). Focusing on the correlation between successive deviations quantifies the tendency of an infant to stay on the same centile. This correlation can be viewed as a measure of feedback, indicating how the initial rate of centile crossing influences the subsequent rate. When successive deviations are positively correlated, individuals tend to cross centiles in the same direction in the 2 periods, either up or down, reflecting an underlying disposition to move away from their initial centile toward a new centile. This is positive feedback.

Conversely, a negative correlation indicates a tendency to cross centiles in opposite directions in successive periods—for example, when growth faltering is followed by catch-up growth (i.e., downward followed by upward centile crossing). In this case, the second deviation partially compensates for the first and tends to shift the growth trajectory back toward the initial centile. Such negatively correlated successive deviations indicate negative feedback.

Thus, the correlations between deviations at different ages inform the nature of centile crossing over time. This leads to several questions. How does the correlation vary with age? Are there ages when weight gain is more or less useful clinically in predicting future deviation? And, extending the question to look at weight deviation over all possible pairs of time intervals, not just adjacent measurement pairs, is the correlation structure informative about the underlying process of infant growth?

The aim of the study was to explore the correlation structure of weight deviation in 2 cohorts of infants measured monthly during the first year of life. One of the cohorts also had measurements continuing up to age 6 y.

## METHODS

### Subjects and measurements

The data came from 2 longitudinal growth studies of infants born in Cambridge, United Kingdom. The Widdowson cohort, assembled by Elsie Widdowson (13), consisted of 1094 Cambridge infants born in 1959–1965 whose routine monthly weights from birth to 12 mo were obtained from the records of child welfare clinics. The maximum number of weights was 13, and all but 6 infants had ≥11 weights (14). In addition, 20 weights were excluded on data cleaning, where the residuals after fitting a SITAR (SuperImposition by Translation And Rotation) growth curve model to the data exceeded ±4 residual SDs (15). Ethical approval was not sought, because at that time it was not required for such secondary data.

The Cambridge Infant Growth Study (CIGS) consisted of 269 infants recruited during 1984–1987, in 4 equally sized sweeps, from lists of Cambridge city mothers booked to deliver in particular months; health visitors excluded a few mothers they felt unsuitable to be included. The infants were weighed by a highly trained auxologist on up to 21 occasions: every 4 wk from birth to 52 wk (14 measurements), every 6 mo from 18 to 36 mo (4 measurements), then annually to 6 y (3 measurements). In addition, all but the first sweep were seen at 15 mo. The oldest ages of measurement for each sweep were 6, 5, 3, and 2 y, respectively. Thus, the first-year measurements were every 4 wk, similar to the monthly Widdowson measurements. Of the 269 infants, all but 14 were followed up after birth and, of these, all 255 had ≥4 measurements and 240 had ≥16 measurements (16). The data had been cleaned previously. Ethical approval was granted by the ethics committees of the Medical Research Council Dunn Nutrition Centre Cambridge and the Cambridge Area Regional Health Authority.

The Widdowson cohort was broadly representative of Cambridge infants in ∼1960, whereas the CIGS families were more selected and of higher social class. In addition, the Widdowson weights were routinely collected, whereas those for CIGS, apart from birth weight, were obtained under research conditions. The sample sizes were chosen to match the available resources.

### Statistical methods

Weights were converted to *z* scores *z_i_* at the *k* design ages *t_i_*, *i* = 1…*k,* based on the WHO 2006 growth standard and reference (17, 18). Deviations in *z* score between pairs of *z_i_* were calculated for each individual, so that for the interval from *t_i_* to *t_j_* (*j* > *i*) the deviation was given by *d_ij_ = z_j_ – z_i_*. In the simplest case, *j = i +* 1; and the deviation was the change in *z* score from one occasion to the next. However, with *k* measurement occasions there were *k*(*k* – 1)/2 possible (*i, j*) measurement pairs, with each deviation indicating how weight *z* score changed over that time interval, corresponding to centile crossing.

Next, the Pearson correlation matrix of the *k*(*k* – 1)/2 deviations was calculated, so that each correlation was that between a pair of deviations, representing the association between centile crossing in the 2 intervals. Each deviation involved 2 *z_i_*; so, for deviations not adjacent in time, the correlation between the deviations involved 4 *z_i_*. **Appendix A** shows that each such correlation is a function of the correlations between pairs of the *z_i_* such that


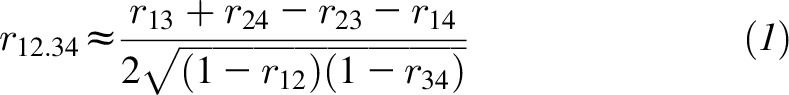


where *r*_12.34_ is the correlation between deviations *d*_12_ and *d*_34_ and *r_ij_* is the correlation between *z_i_* and *z_j._*

Pairs of deviations adjacent in time share a common measurement (i.e., *d*_12_ and *d*_23_ both involve *z_2_*), and the shared measurement error biases the 2 deviations in opposite directions. This, in turn, biases downward the correlation between the deviations, which, expressed in terms of the *z* score correlations, is given (see Appendix A) by the following:


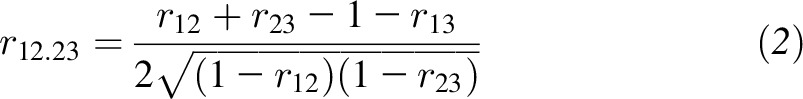


The bias could be avoided by using nonadjacent time intervals (1), hence making the correlation more positive; however, if growth feedback were operating, either positively or negatively, the time gap would also, by definition, weaken the feedback correlation, attenuating it toward zero. The use of longer time intervals would also reduce measurement error, although, again, this could attenuate the feedback correlation.

A completely different analytic approach was to consider deviations over all possible time intervals (i.e., not just adjacent intervals) and to identify strongly (positively or negatively) correlated pairs of deviations. Note that for this analysis, pairs of deviations with overlapping time intervals (e.g., *d*_14_ and *d*_23_) were excluded.

In principle, the correlations would be affected by the measurement ages differing slightly from the target ages. However, it turned out that adjusting for this source of variability rarely altered the correlations by >0.01, so it was ignored.

The clinical impact of growth feedback was derived as follows. The regression equation corresponding to the correlation *r*_12.23_ is the regression of the second deviation on the first:


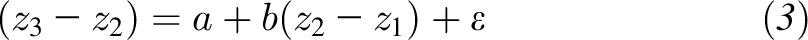


where *a* is the intercept, *b* the regression coefficient, and ε the error term. Coefficient *b* and correlation *r*_12.23_ are equally significant by definition and, in practice, were very similar in value because the variances of the 2 deviations were similar. Equation *3* can be rearranged to give the algebraically identical equation that predicts the future *z* score (*z*_3_) from the recent deviation (*z*_2_ – *z*_1_) and the current *z* score (*z*_2_):


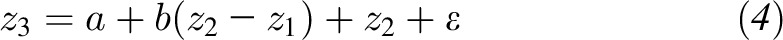


Now, Equation *4* can be generalized by regressing *z*_3_ on (*z*_2_ – *z*_1_) and *z*_2_, estimating coefficient *c* for *z*_2_:


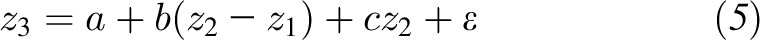


It turned out that Equation *5* fit better than Equation *4*, and *c* was usually <1 (i.e., adjusting for regression to the mean). So, if *r*_12.23_ (and hence *b*) was significant it was worthwhile to use the deviation (*z*_2_ – *z*_1_) as well as the current *z* score (*z*_2_) to predict the future *z* score (*z*_3_). The size of *b* indicated how much predicted *z*_3_ changed among infants with the same *z_2_*, given a unit increase in (*z*_2_ – *z*_1_). Intercept *a* showed how heavy the infants were compared with the WHO standard.

## RESULTS

### Data summary

Summary statistics for the ages of measurement, weight *z* scores, adjacent time intervals, and weight *z* score deviations by age group for the 2 cohorts are shown in **Table 1** (Widdowson) and **Table 2** (CIGS). The mean ages and time intervals were close to the design values. Up to 12 mo, the SD of the time interval was ∼0.4 wk for CIGS and ∼0.3 mo (i.e., 3 times larger) for Widdowson, so the Widdowson measurements were less precisely timed, which could have attenuated their deviation correlations compared with CIGS.

**TABLE 1:** Weight *z* score and *z* score deviation by age: Widdowson data^1^

	*n*	Age, mo	Weight *z* score	Time interval, mo	Weight *z* score deviation
Age group, mo					
0	1091	0.0 ± 0.0	0.1 ± 1.1	—	—
1	1040	1.0 ± 0.2	−0.3 ± 0.9	—	—
2	1080	2.0 ± 0.2	−0.3 ± 1.0	—	—
3	1079	3.0 ± 0.2	−0.2 ± 0.9	—	—
4	1072	4.0 ± 0.2	0.1 ± 0.9	—	—
5	1071	5.0 ± 0.2	0.3 ± 0.9	—	—
6	1065	6.0 ± 0.3	0.4 ± 0.9	—	—
7	1054	7.0 ± 0.3	0.5 ± 0.9	—	—
8	1043	8.0 ± 0.3	0.6 ± 0.9	—	—
9	1009	9.0 ± 0.3	0.7 ± 0.9	—	—
10	969	10.0 ± 0.3	0.7 ± 0.9	—	—
11	913	11.0 ± 0.4	0.7 ± 0.9	—	—
12	852	12.0 ± 0.4	0.7 ± 0.9	—	—
Age interval group, mo					
0–1	1037	—	—	1.0 ± 0.2	−0.4 ± 0.6
1–2	1035	—	—	1.0 ± 0.2	0.0 ± 0.4
2–3	1066	—	—	1.0 ± 0.2	0.2 ± 0.4
3–4	1058	—	—	1.0 ± 0.3	0.2 ± 0.3
4–5	1051	—	—	1.0 ± 0.3	0.2 ± 0.3
5–6	1043	—	—	1.0 ± 0.3	0.1 ± 0.2
6–7	1026	—	—	1.0 ± 0.3	0.1 ± 0.2
7–8	1003	—	—	1.0 ± 0.3	0.1 ± 0.2
8–9	961	—	—	1.0 ± 0.3	0.1 ± 0.2
9–10	890	—	—	1.0 ± 0.3	0.0 ± 0.2
10–11	807	—	—	1.0 ± 0.3	0.0 ± 0.2
11–12	698	—	—	1.0 ± 0.3	0.0 ± 0.2

1 Values are means ± SDs unless otherwise indicated.

**TABLE 2:** Weight *z* score and *z* score deviation by age group: CIGS data^1^

	*n*	Age, wk	Weight *z* score	Time interval, wk	Weight *z* score deviation
Age group					
0 wk	269	0.0 ± 0.0	0.1 ± 1.0	—	—
4 wk	253	4.0 ± 0.3	−0.2 ± 0.9	—	—
8 wk	254	8.1 ± 0.2	−0.2 ± 0.9	—	—
12 wk	255	12.0 ± 0.2	−0.3 ± 0.9	—	—
16 wk	253	16.0 ± 0.3	−0.2 ± 0.9	—	—
20 wk	251	20.0 ± 0.3	−0.1 ± 0.9	—	—
24 wk	251	24.0 ± 0.3	0.0 ± 0.9	—	—
28 wk	247	28.1 ± 0.3	0.0 ± 0.9	—	—
32 wk	251	32.1 ± 0.3	0.1 ± 0.9	—	—
36 wk	250	36.0 ± 0.3	0.1 ± 0.9	—	—
40 wk	247	40.0 ± 0.3	0.2 ± 0.9	—	—
44 wk	245	44.0 ± 0.3	0.2 ± 0.9	—	—
48 wk	246	48.1 ± 0.4	0.2 ± 0.9	—	—
52 wk	247	52.1 ± 0.4	0.2 ± 0.9	—	—
15 mo	183	65.4 ± 0.8	0.3 ± 0.9	—	—
18 mo	240	78.2 ± 1.2	0.2 ± 0.9	—	—
24 mo	239	105 ± 1.5	0.1 ± 0.9	—	—
30 mo	187	131 ± 2.2	0.1 ± 0.9	—	—
3 y	187	158 ± 1.9	0.0 ± 0.9	—	—
4 y	117	210 ± 2.5	−0.1 ± 0.9	—	—
5 y	115	262 ± 1.6	−0.1 ± 0.9	—	—
6 y	52	314 ± 1.1	0.0 ± 0.9	—	—
Age interval group					
0–4 wk	253	—	—	4.0 ± 0.3	−0.3 ± 0.6
4–8 wk	252	—	—	4.0 ± 0.3	−0.1 ± 0.4
8–12 wk	254	—	—	4.0 ± 0.3	0.0 ± 0.3
12–16 wk	253	—	—	4.0 ± 0.3	0.1 ± 0.3
16–20 wk	251	—	—	4.0 ± 0.4	0.1 ± 0.3
20–24 wk	250	—	—	4.0 ± 0.4	0.1 ± 0.2
24–28 wk	246	—	—	4.0 ± 0.4	0.0 ± 0.2
28–32 wk	247	—	—	4.0 ± 0.4	0.1 ± 0.2
32–36 wk	250	—	—	4.0 ± 0.3	0.0 ± 0.2
36–40 wk	247	—	—	4.0 ± 0.4	0.1 ± 0.2
40–44 wk	245	—	—	4.0 ± 0.4	0.0 ± 0.2
44–48 wk	243	—	—	4.0 ± 0.4	0.0 ± 0.2
48–52 wk	246	—	—	4.0 ± 0.5	0.0 ± 0.2
52 wk–15 mo	182	—	—	13.3 ± 0.8	0.0 ± 0.3
15–18 mo	180	—	—	13.0 ± 1.2	0.0 ± 0.3
18–24 mo	238	—	—	26.6 ± 1.7	−0.1 ± 0.3
24–30 mo	187	—	—	26.3 ± 2.4	−0.1 ± 0.3
30 mo–3 y	185	—	—	26.3 ± 2.4	0.0 ± 0.3
3–4 y	117	—	—	52.1 ± 2.9	0.0 ± 0.3
4–5 y	112	—	—	52.3 ± 3.2	0.0 ± 0.3
5–6 y	52	—	—	51.6 ± 2.0	0.0 ± 0.2

1 Values are means ± SDs unless otherwise indicated. CIGS, Cambridge Infant Growth Study.

The mean weight *z* score decreased sharply during the first month, and in the Widdowson cohort it then increased steadily to 0.7 at 12 mo; for CIGS, the increase was slower, to 0.3 at 15 mo, followed by a drift back to zero (see reference 14 for details). In both cohorts, the SD of the weight *z* score was 0.9 at most ages, which was slightly less than the expected value of 1.0. The mean *z* score deviation was negative for the first month but near zero thereafter, whereas the SD of the deviation decreased from 0.6 at 1 mo to 0.2 at 5 mo, and then increased to 0.3 from 1 y onward in CIGS. Thus, on average, infants did not cross centiles—those crossing upward balanced those crossing downward—and the range of centile crossing decreased substantially from birth to 5 mo, then remained constant until 12 mo when it increased slightly due to the longer time intervals.

### Correlations of adjacent short-term deviations

**FIGURE 1:**
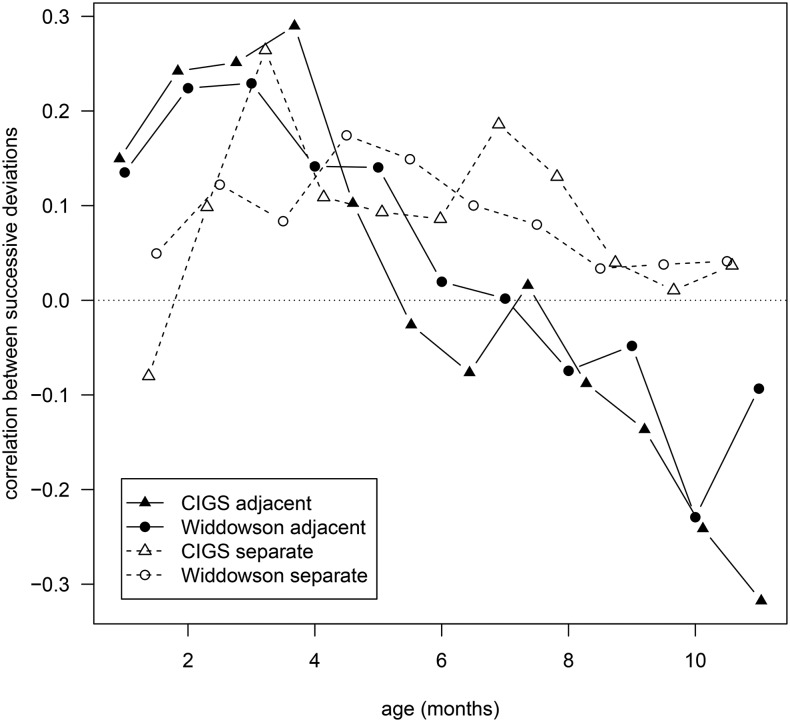
Correlations by age between pairs of weight *z* score deviations measured over 1 mo (Widdowson) or 4 wk (CIGS), where the corresponding time periods are either adjacent or separated by 1 mo or 4 wk, respectively. Correlations are plotted at the midage of the 2 periods. The sample sizes for each correlation are given in Tables 1 and 2. CIGS, Cambridge Infant Growth Study.

**Figure 1** gives the correlations between pairs of weight *z* score deviations during the first year, each measured over 1 mo in Widdowson and 4 wk in CIGS. The solid points and lines are correlations for adjacent deviations, whereas the open points and dashed lines are correlations for deviations separated by 1 time interval (i.e., 1 mo or 4 wk). Each correlation is plotted at the midage of the pair of intervals.

**FIGURE 2:**
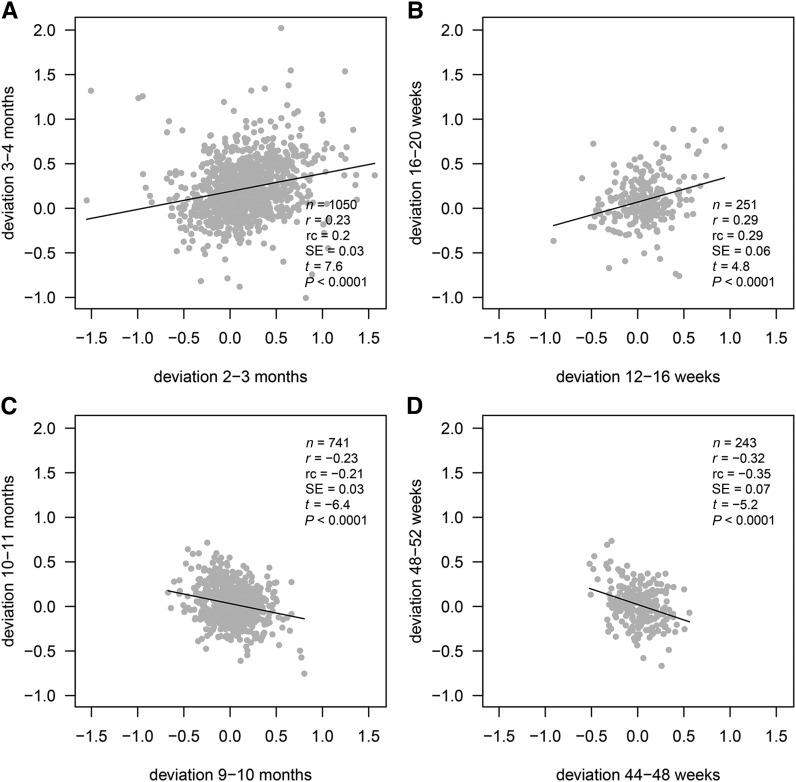
Scatterplots corresponding to the extreme correlations in Figure 1: Widdowson maximum (A), CIGS maximum (B), Widdowson minimum (C), and CIGS minimum (D), with fitted regression lines and summary statistics. CIGS, Cambridge Infant Growth Study; rc, regression coefficient.

The patterns of change in correlation with age were strikingly similar in the 2 cohorts. The correlations for adjacent deviations (solid lines) increased from ∼0.15 at 1 mo to a zenith of ∼0.25 at 3–4 mo. Thus, there was clear positive feedback up to 4 mo (infants growing faster or slower in the first interval tending to grow faster or slower in the second), and the degree of positive feedback increased with age. **Figure 2**A and B shows scatterplots corresponding to the largest correlation in each cohort, with the use of the same axis scales and confirming the absence of outliers. Note that the variability was appreciably less for CIGS than for Widdowson, which reflects the higher quality measurements.

After 4 mo, the correlations in Figure 1 started to decrease, ending up below −0.2 at 10 mo (Widdowson) and 11 mo (CIGS), indicating marked negative feedback (*P-*trend < 0.0001). Figure 2C and D shows the corresponding scatterplots, with the variability being less than earlier in infancy (Figure 2A, B). Note also in Figure 1 that the Widdowson correlation bounced back up to −0.1 at 11 mo, so that the 10-mo correlation was the nadir. For CIGS, the 48-wk correlation may have been the nadir, but the 52-wk correlation could not be calculated because there was no 56-wk measurement. However the correlation of 48–52 wk with 52 wk–15 mo was −0.04, which implies that 48–52 wk compared with 52–56 wk would also have been close to zero, making 48 wk the nadir.

Despite the modest sizes of ±0.2 to 0.3—the variance explained rarely exceeded 10%—these extreme correlations were highly significant and the pattern with age was entirely consistent [correlations of ±0.15 (Widdowson) or ±0.25 (CIGS) were significant at *P* = 0.0001]. The CIGS correlations tended to be larger than those for Widdowson in absolute terms.

### Clinical importance of correlated short-term deviations

**FIGURE 3:**
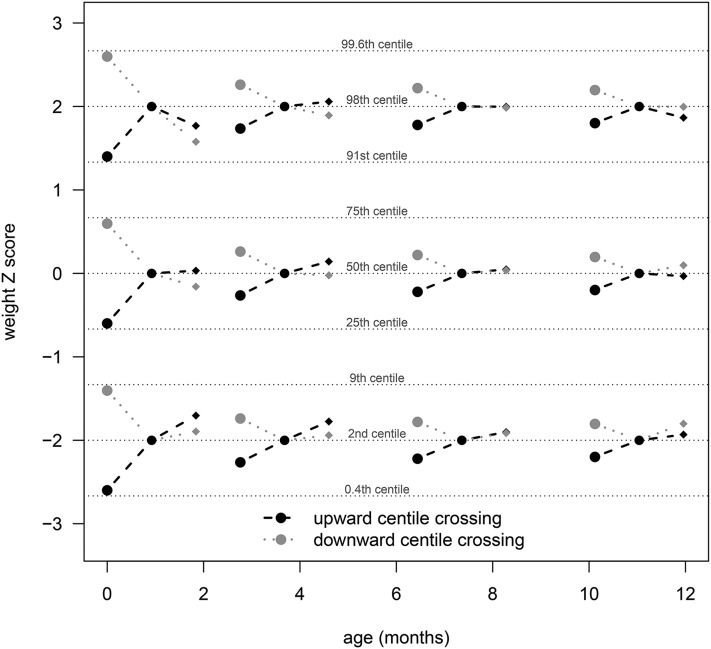
Weight *z* score chart showing predicted weight (diamonds) for contrasting patterns of recent centile crossing (circles), for infants at particular ages and weight centiles, based on the CIGS data and Equation *5* (see text for details). CIGS, Cambridge Infant Growth Study.

Clinically, the significant correlations in Figure 2 indicate that, at those ages, an infant’s future *z* score would be best predicted by both their current *z* score and their recent *z* score deviation. **Table 3** summarizes the corresponding regressions (5), and **Figure 3** shows how they affect clinical prediction at 4 different ages by using the CIGS data. The weight *z* score chart in Figure 3 shows centiles spaced two-thirds of a *z* score apart (19) and hypothetical light (second centile), median (50th centile), and heavy (98th centile) infants at ages 4, 16, 32, and 48 wk. Each age-centile combination contrasts patterns of upward (solid dashed lines) and downward (gray dotted lines) centile crossings. For each example, the previous, current, and predicted weights are plotted, showing the impact of recent upward compared with downward centile crossing (circles) on predicted weight (diamonds), in infants of the same current weight (solid circles), for different ages and weight centiles.

**TABLE 3:** Multiple regressions of future weight on recent deviation and current weight Equation *5* by cohort and age (Figure 2): *z* score scale^1^

Cohort	Current age	*r*_12.23_^2^	Recent deviation, *b* ± SE	Current weight, *c* ± SE	Intercept, *a* ± SE
Widdowson	3 mo	0.23	0.24 ± 0.03	1.15 ± 0.03	0.17 ± 0.01
CIGS	16 wk	0.29	0.31 ± 0.06	0.96 ± 0.02	0.06 ± 0.02
Widdowson	10 mo	−0.23	−0.20 ± 0.03	0.78 ± 0.03	0.05 ± 0.01
CIGS	48 wk	−0.32	−0.33 ± 0.07	0.95 ± 0.01	0.03 ± 0.01

1 Each row represents 1 regression model. *a*, *b*, and *c* are coefficients in Equation *5*. CIGS, Cambridge Infant Growth Study.

2 Correlation of deviations *d*_12_ and *d*_23_ Equation *2*.

In Figure 3, the amount of centile crossing at each age is set at ±1 SD (i.e., contrasting the 16th and 84th deviation centiles; see Table 2), which was largest at 0–4 wk and progressively smaller at later ages. The effect of centile crossing on predicted weight is the difference between the black and gray diamonds, amounting to a quarter of a channel width at 8 and 20 wk, zero at 36 wk, and minus one-fifth of a channel width at 52 wk. This effect is the same on all 3 weight centiles, and there is a separate effect of regression to the mean as seen by contrasting the 2nd and 98th centiles.

### Correlations of nonadjacent short-term deviations

The correlations for 1-mo (4-wk) deviations separated by 1 mo (4 wk) are shown in Figure 1 by the open circles and dashed lines. They were smaller than for adjacent deviations ≤4 mo of age and remained small and positive thereafter. If the measurement errors had been large enough to bias the correlations, the dashed lines would have been shifted downward compared with the solid lines. The fact that they were not indicates that measurement error had little effect compared with the bias caused by introducing a time gap between deviations. In addition, the results for the 2 cohorts were essentially identical despite the differences in measurement quality. Thus, the role of measurement error could be discounted. The fact that the correlations were modestly positive and fairly constant over time may reflect longer-term tracking superimposed on the short-term feedback effects.

### Correlations of longer-term deviations

The alternative way to study the correlation structure was to search each cohort’s deviation correlation matrix for extreme positive or negative values. For CIGS, the matrix was restricted to the first 3 sweeps (i.e., measurements from birth to 3 y) to ensure adequate numbers (*n* ≥ 180). The 19 measurement ages from birth to 3 y gave 171 deviations (18 adjacent occasions, 17 two occasions apart, 16 three apart, …, 1 over 3 y). They led to 171(171 − 1)/2 or 14,535 correlations between pairs of deviations, of which one-third (4845) were for nonoverlapping time periods. The corresponding numbers for Widdowson, with 13 measurements, were 78 deviations and 3003 correlations, of which 1001 were nonoverlapping.

**Tables 4** and **5** show the largest positive correlations, and **Tables 6** and **7** the largest negative correlations, in the 2 cohorts. Table 4 shows the 7 largest positive Widdowson correlations out of the 1001, all similar in size, all for adjacent periods, and all highly significant (*P* < 0.0001). The midages of the deviations all fell in the range of 2–6 mo.

**TABLE 4:** Widdowson data: 7 largest correlations between deviations^1^

	Age interval, mo		
Age interval, mo	1–2	3–4	3–5
2–3	0.22	0.23	0.21
1–3	—	0.22	—
2–4	0.23	—	—
4–6	—	0.22	—
5–8	—	—	0.22

1 The correlations show that pairs of deviations in the period 2–5 mo are strongly correlated. Correlations (all *P* < 0.0001) are based on a median of 1020 infants.

**TABLE 5:** CIGS data: 8 largest correlations between deviations^1^

	Age interval, wk		
Age interval, wk	8–12	12–16	8–16
16–20	—	0.29	0.35
12–20	0.32	—	—
16–24	—	0.27	0.31
12–24	0.29	—	—
16–28	—	0.30	0.30

1 The correlations show that deviations between 8 and 16 wk are strongly correlated with adjacent deviations between 16 and 28 wk. Correlations (*P* < 0.0001) are based on a median of 250 infants. CIGS, Cambridge Infant Growth Study.

**TABLE 6:** Widdowson data: 4 largest negative correlations between deviations^1^

	Age interval, mo	
Age interval, mo	9–10	0–2
10–11	−0.23	—
4–8	—	−0.20
4–10	—	−0.21
4–11	—	−0.21

1 All correlations but the first show that the 0–2 mo deviation is strongly inversely correlated with deviations later in the year. Correlations (*P* < 0.0001) are based on a median of 906 infants.

**TABLE 7:** CIGS data: 9 largest negative correlations between deviations up to 3 y^1^

	Age interval		
Age interval	12–32 wk	8–32 wk	8–36 wk
52 wk–3 y	−0.44	−0.46	−0.45
48 wk–3 y	−0.44	−0.46	−0.45
44 wk–3 y	−0.44	−0.46	−0.44

1 All correlations show that deviations at ∼12–32 wk are strongly inversely correlated with deviations at 1–3 y. Correlations (*P* < 0.0001) are based on a median of 184 infants. CIGS, Cambridge Infant Growth Study.

Table 5 shows the 8 largest positive CIGS correlations up to 3 y (*P* < 0.0001). All were for adjacent periods, of 4–12 wk duration, and with midages of 12–16 wk. Therefore, pairs of deviations within the age range 8–28 wk (2–6 mo) exhibited strong positive feedback, whereas there were no large positive correlations involving measurements after 28 wk. Note that these CIGS correlations were appreciably larger than those for Widdowson in Table 4 (median of 0.30 compared with 0.22).

Table 6 shows the 4 largest negative Widdowson correlations, in which the pattern was very different. The most negative correlation was −0.23 for deviations >9–10 compared with 10–11 mo (i.e., adjacent and centered on 10 mo; the nadir in Figure 1 and see Figure 2C). The other 3 correlations were for the 0–2 mo deviation with deviations from 4 mo onward, in which the pairs of deviations were separated by 2 mo.

Table 7 shows the 9 largest negative CIGS correlations up to 3 y. Deviations for ∼12–32 wk were strongly inversely correlated with deviations for ∼1–3 y, indicating marked negative feedback.

## DISCUSSION

The results showed a complex correlation structure of monthly weight centile crossing in infancy, with a peak at 3–4 mo (correlation: 0.3) and a trough at 9–10 mo (correlation: −0.3), with the same findings emerging in 2 cohorts of rather different design. Thus, upward-downward centile crossing in the first few months tends to be followed by upward-downward centile crossing the following month, whereas in the second half of the first year the reverse is true: upward-downward centile crossing is likely to be followed by downward-upward centile crossing. Despite the modest size of the correlations, they are consistent both within and between cohorts and are highly significant. This suggests that the pattern is genuine.

The correlations can be viewed as growth feedback, being positive in the early months and negative later. Positive feedback is intrinsically unstable, implying continued centile crossing, so it makes sense biologically to view the period of positive feedback as a critical window that closes after the early months, and it then transitions to negative feedback which is an intrinsically more stable state. This pattern helps explain why many studies reported associations between early growth variability and later overweight status (5, 6, 8, 9, 20). The underlying mechanisms may involve several hormones and their receptors (21, 22).

The correlation structure has 3 particular features of note. The first is the positive pattern for centile crossing from birth to 6 mo; the second is the negative pattern from 6 to 12 mo, with a nadir at 10–11 mo; and the third is the negative correlation between early life and later childhood.

The positive correlations up to 6 mo are clearly a measure of the individual’s determination to grow; infants are shifting from their birth centile to another preferred centile, and growth deviations over a given month tend to be in the same direction the following month. This also applies over longer time periods: all of the large positive correlations in Tables 4 and 5 are for adjacent time intervals of up to 3 mo. This urge to cross centiles is likely to be driven at least in part by genetic and environmental contributions from paternal and maternal weight and height (23). It peaks at ∼12–16 wk, and then fades away and goes into reverse as the correlation becomes negative (Figure 1). Thus, past 6 mo, infants are less likely to shift to a new centile and are more likely to stay near their initial centile.

The most negative correlations in the first year occur at 10 mo for Widdowson and at 48 wk (11 mo) for CIGS. The Widdowson cohort is therefore slightly earlier, but its underlying growth pattern is accelerated compared with CIGS, with less breastfeeding and greater upward centile crossing during the first year (14). Thus, the 2 timings may be more similar in terms of developmental age. In addition, the Widdowson correlation 1 mo after the nadir is close to zero, and that for CIGS is probably very small (although the absence of a 56-wk measurement makes it uncertain). Thus, at this point in late infancy, the strongly negative correlation and hence negative feedback that has kept infants near the same centile suddenly eases, and their growth deviation in the following month is essentially uncorrelated with what has gone before. This suggests that they have transitioned to a new phase of growth.

Karlberg’s ICP (infancy-childhood-puberty) height growth model (24) proposes such a transition from infant to childhood growth toward the end of the first year, which is visible as a small but consistent change in length velocity (i.e., centile crossing) (25, 26). So it is plausible that the large negative correlation followed by zero correlation reflects the ICP infant-childhood transition, albeit for weight rather than height. There is also evidence from cross-sectional data of a change in growth pattern at this time. The LMS method (27), which is used to construct growth references, estimates the CV of the measurement as a function of age, a plot called the S curve. For weight and other measurements, including length and head circumference, the S curve falls steeply after birth to a nadir near to the end of the first year (i.e., at the same time as the nadir here), and it then rises again (28–30). Both patterns are consistent with growth shifting to a new regulatory regimen at this time. Before the nadir, centile crossing allows infants to shift to their preferred centile, and as a result the population CV falls as the distribution narrows. Then, after the nadir, children start shifting to a new centile, and the CV rises again.

The third correlation pattern of note is the cluster of large negative correlations seen in Tables 6 and 7, between deviations in early life (0–2 mo for Widdowson and 2–5 mo for CIGS) and deviations later in infancy and childhood (4–10 mo for Widdowson and 1–3 y for CIGS). This is the situation with Equation *1*, in which a large negative correlation results when *r*_13_ is similar to *r*_14_ but *r*_23_ is appreciably larger than *r_24_*, as occurs when age 1 is early, ages 2 and 3 are intermediate, and age 4 is relatively late.

An important practical question is the extent to which knowledge of this correlation pattern might benefit the management of infants in a clinical setting. Does it improve the prediction of future weight? The answer is a qualified yes; Figure 3 shows that recent centile crossing can affect predicted weight by up to one-quarter of a channel width, but the size and direction of the effect are strongly age-dependent. So, when a practitioner is examining an infant who has recently crossed centiles, she or he needs to know if the pattern might be due to previous centile crossing. Figure 3 shows that, in early infancy, centile crossing predicts further centile crossing in the same direction; in late infancy, it predicts it in the opposite direction; and in midinfancy it is not predictive.

It is possible that infant feeding mode affects the correlation pattern, particularly in the first 6 mo, because this is the period when growth is under nutritional control. However, the CIGS cohort was of appreciably higher social class, with higher rates of breastfeeding overall, than the Widdowson cohort, and yet the 2 correlation patterns are virtually identical, which suggests that breastfeeding is not a major influence.

Another question relates to generalizability: to what extent do the findings apply to other populations—for example, where growth is more disrupted by infection and malnutrition? An intriguing possibility is that critical windows in the sensitivity of growth to nutrition may vary in duration between populations, which may, in turn, account for contrasting associations between early weight gain and later body composition between high-income and low- and middle-income populations (31, 32). The approach described here might help to test this hypothesis. Interestingly, in a study in Brazilian infants, there was no association between weight gain from 6 to 12 mo and either fat or lean mass in later childhood (6), which supports the notion of late infancy as a period in which the mechanism of growth regulation shifts from nutrition to growth hormone.

Finally, the data presented are relatively old, and there may be concern that the findings do not apply to more recently born infants. However, there were few differences in weight gain between white English infants from the ALSPAC (Avon Longitudinal Study of Parents and Children) cohort born in 2001–2002 and the Millennium cohort born 10 y later (33), which suggests that infant growth has not been changing materially.

A strength of the study is that, in both the Widdowson and CIGS cohorts, the design time intervals between measurements in the first year were constant (i.e., 1 mo and 4 wk, respectively). This is an unusual design, and without it the age trends in the correlation could not have been explored in the same way. A limitation is that there was not a 56-wk measurement in CIGS, which would have provided stronger evidence for a nadir in correlation at 48 wk.

In conclusion, the study shows that, in infancy, weight *z* score deviations over a 1-mo period are correlated with deviations the following month. The magnitude of the correlation changes with age, from strongly positive at 4–5 mo to strongly negative at 10–11 mo. This tracking of centile crossing over time represents feedback, whereby centile crossing in 2 successive months tends to be in the same direction up to 5 mo of age (positive feedback) and in the opposite direction later in infancy (negative feedback). In clinical terms, the feedback means that recent centile crossing needs to be interpreted in the context of any previous centile crossing.

## APPENDIX A

Consider *z* scores *z_i_* for ages *t_i_*, *i* = 1…4, where corr(*z_i_,z_j_*) = *r_ij_*. Also consider *z* score deviations* d_ij_* = *z_j_* − *z_i_* for the time interval *t_i_* to *t_j_*. On the assumption that var(*z_i_*) = *V* for all *i* (where *V* ≈ 1), the correlation between pairs of nonadjacent deviations *d*_12_ and *d*_34_ is given by *r*_12.34_, where


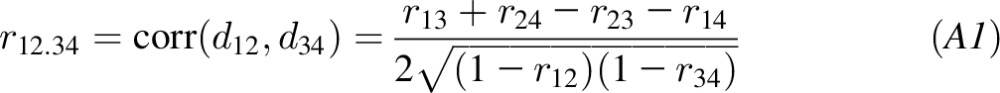


Equation *A1* arises as follows: the covariance between deviations is


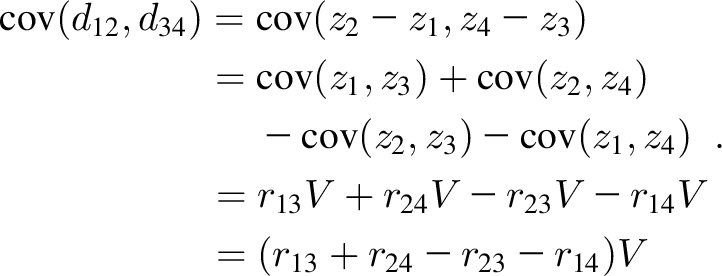


The variance of each deviation is of the form


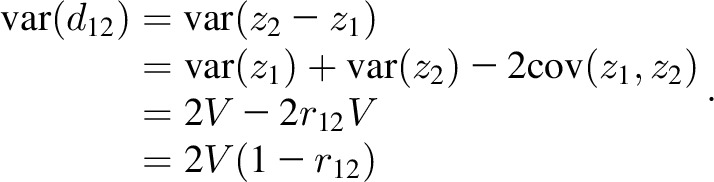


The correlation coefficient is then defined as


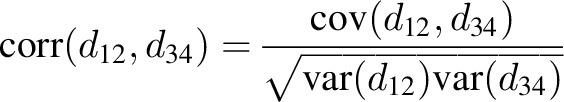


where the *V*s cancel out, so that Equation *A1* applies even when 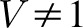.

When the 2 deviations are adjacent in time, *z_3_* = *z_2_* and *r_23_* = 1. Renumbering occasions 3 and 4 as 2 and 3, the correlation in Equation *A1* becomes